# Hemolysis by Saponin Is Accelerated at Hypertonic Conditions

**DOI:** 10.3390/molecules28207096

**Published:** 2023-10-15

**Authors:** Boyana Paarvanova, Bilyana Tacheva, Gergana Savova, Miroslav Karabaliev, Radostina Georgieva

**Affiliations:** 1 Department of Physics and Biophysics, Faculty of Medicine, Trakia University, 11 Armeiska, 6000 Stara Zagora, Bulgaria; bilyana.tacheva@trakia-uni.bg (B.T.); gergana.savova@trakia-uni.bg (G.S.); miroslav.karabaliev@trakia-uni.bg (M.K.); 2 Institute of Transfusion Medicine, Charité-Universitätsmedizin Berlin, Charitéplatz 1, 10117 Berlin, Germany

**Keywords:** saponin, erythrocytes, hemolysis, hypertonic conditions, hemolytic assay

## Abstract

Saponins are a large group of organic amphiphilic substances (surfactants) mainly extracted from herbs with biological activity, considered as one of the main ingredients in numerous remedies used in traditional medicine since ancient times. Anti-inflammatory, antifungal, antibacterial, antiviral, antiparasitic, antitumor, antioxidant and many other properties have been confirmed for some. There is increasing interest in the elucidation of the mechanisms behind the effects of saponins on different cell types at the molecular level. In this regard, erythrocytes are a very welcome model, having very simple structures with no organelles. They react to changing external conditions and substances by changing shape or volume, with damage to their membrane ultimately leading to hemolysis. Hemolysis can be followed spectrophotometrically and provides valuable information about the type and extent of membrane damage. We investigated hemolysis of erythrocytes induced by various saponin concentrations in hypotonic, isotonic and hypertonic media using measurements of real time and end-point hemolysis. The osmotic pressure was adjusted by different concentrations of NaCl, manitol or a NaCl/manitol mixture. Unexpectedly, at a fixed saponin concentration, hemolysis was accelerated at hypertonic conditions, but was much faster in NaCl compared to mannitol solutions at the same osmotic pressure. These findings confirm the colloid-osmotic mechanism behind saponin hemolysis with pore formation with increasing size in the membrane.

## 1. Introduction

Saponins are a large group of organic substances extracted from plants and some marine species [1]. They have a complex and varied structure and belong to the surfactants due to their amphiphilic nature, which also determines their biological activity. Being amphiphilic, the molecules of saponins have two moieties, a hydrophilic one and lipophilic one. The hydrophilic moiety is also called the glycone part of the saponin molecule and consists of one or more sugars. The lipophilic moiety is called aglycone, as well as sapogenin, and may be a triterpenoid and/or steroid. Depending on the latter, the saponins are usually classified as a triterpenoid or steroid [2,3].

It is considered that saponins are one of the main ingredients in numerous medicines used in folk medicine since ancient times [4,5]. Currently, the properties of many different saponins have been established. They are used in the food industry, cosmetics and pharmacy. Some of them have been found to have anti-inflammatory [6,7,8], antifungal [9,10,11], antibacterial [10,12], antiparasitic [13,14], antitumor [15,16,17], antiviral [18,19,20], antioxidant [21] and many other properties. Their use as food additives to decrease blood cholesterol levels [22], as well as their application as drug delivery systems [23] are also under investigation.

These unique properties of saponins impose the study of their effects on different cell types in order to elucidate the mechanisms behind them at the molecular level. Erythrocytes, the red blood cells (RBCs), are a very convenient object to study the influence of any drug on the cell membrane due to their simple structure. These cells are without any organelles and their cytoskeleton is reduced to a spectrin net below the lipid bilayer. Additionally, they are easily isolated in large quantities as a pure monoculture.

Under normal physiological conditions, erythrocytes have a biconcave shape (discocytes). Depending on the conditions of the environment, they can change their shape to spherical with spicules on the surface (echinocytes), to a cup-like form with the membrane deformed inwards on one side (stomatocytes) or to an almost spherical shape and increased volume (spherocytes). The induction of such shape modifications is accompanied by disturbance of the RBC membrane and finally can lead to hemolysis, represented by the release of hemoglobin into the outside medium. Generally, the stability of membranes can be affected by many environmental factors like temperature, osmotic pressure, ionic strength or pH. In hypotonic hemolysis, for example, the RBCs swell, resulting in conformational and/or associative changes of the anion-transport protein (band 3) in the erythrocyte membrane, formation of holes and subsequent hemolysis [24].

The presence of surfactants, including saponins or other membrane active drugs at a certain concentration, can also cause hemolysis. The main supposed mechanisms behind include hemolysis by lipid solubilization and hemolysis after the formation of pores and larger membrane defects [25,26]. However, the type of the applied surfactant and its concentration determines the specific predominant mechanism of hemolysis.

Several stages of hemolysis induced by the simultaneous exposure to a hypotonic suspension medium and detergent were described [27]. By adsorbing to the membrane, the detergent disrupts the assembly of phospholipids and alters the interactions between phospholipids and integral proteins, leading to an increased ionic permeability of the membrane. As a result, K${}^{+}$ ions diffuse out of and Na${}^{+}$ into the cell. Simultaneously, water is transported into the cell balancing the difference in osmotic pressure, the cells swell, change their shape from discocytic to spherocytic and finally lyse.

Saponins have been found to damage the erythrocyte membrane by forming insoluble complexes with cholesterol [28,29,30,31,32]. As a result, membrane defects appear most often in the form of specific pores in the membrane [33] with a diameter of 40–50 Å [31,34]. Baumann et al. [35] reported that during the permeabilization of erythrocytes with saponins, changes of membrane proteins were also observed—the aggregation of band 3 proteins and dissociation of submembrane cytoskeleton junctions from integral proteins. All these changes in the erythrocyte membrane led to pore formation and hemolysis, most likely by a colloid-osmotic mechanism [12]. Abe et al. [36] found that at small concentrations saikosaponins stabilize rat erythrocytes against hypotonic hemolysis.

There are three main groups of factors that affect the process of hemolysis by saponins. The first one is related to the type of the saponin and its chemical structure. It is known that some saponins induce hemolysis while other do not and in this respect there is a classification of the saponins as hemolytic and nonhemolytic [37]. Ondevilla et al. reported that the number of sugars in the saponoin molecule dictates the extent of membrane disruption [38]. Chen et al. also reported about a chemically modified saponin that keeps its antitumor activity but avoids the hemolytic problem [39].

The second group of factors affecting the process of hemolysis is related to the content and phase state of the erythrocyte membrane. By using molecular dynamics simulations, Lin et al. [40] showed that dioscin (a type of saponin) first penetrates into the lipid bilayer, moves towards and accumulates in the lipid raft micro-domain, and complexes with cholesterol therein. These authors suggested that this would destabilize the lipid raft and cause severe curvature of the lipid bilayer, eventually leading to the hemolysis of red cells [40]. Garza et al. [41] demonstrated by model membrane systems that the adsorption of the saponin in the lipid bilayer is more favorable in the liquid-disordered phase Ld compared to the liquid-ordered phase Lo. Orczyk et al. [42] also used a model membrane system and showed that the effect of saponins on the lipid monolayer is related to the amount of cholesterol and that the high sterol content in an erythrocyte cell membrane (47%) is probably responsible for their high affinity to saponins. They found that the incorporation of the saponin in the Langmuir lipid monolayer results in an increase in the surface pressure, which is in accordance with the suggestions by other authors [27] that adsorption of surfactants in the outer monolayer of erythrocytes increases its area, which leads to the formation of echinocytes.

The third group of factors affecting the process of hemolysis by surfactants is the ionic content and ionic strength of the medium [25]. Different types of charged detergents cause different degrees of hemolysis depending on the concentration of ions in the medium. With increasing ionic strength, the rate of hemolysis induced by Dodecyltrimethylammonium bromide (a quaternary ammonium cation) decreases, and by Sodium Dodecyl Sulfate (negatively charged) increases. According to Shalel et al. [25], this is caused by the change in the Debye thickness of the electric double layer around the erythrocyte membrane. As the ionic strength increases, the thickness of the electric double layer decreases, reducing the net negative charge on the surface of the erythrocytes. This weakens the electrostatic interactions between the charged detergent molecules and the erythrocyte membrane. Positively charged detergents are less attracted, the amount adsorbed in the membrane decreases, which leads to a lower rate of hemolysis. In contrast, the reduced repulsion of negatively charged detergents allows the incorporation of a higher amount into the membrane, causing a higher degree of structural disturbance and consequently a higher rate of hemolysis.

Most saponins have strong cytotoxic and hemolytic activity, which makes their direct intravenous application difficult [43]. However, their useful antitumor, antibacterial, antiviral and immunomodulating properties are highly desired for more effective treatments in numerous diseases. Investigations of the factors influencing the speed and the rate of hemolysis caused by saponins could contribute to the optimization of their application using a proper and specifically composed solution for each individual case.

In our work, we compared the process of hemolysis induced by saponin in hypotonic, isotonic and hypertonic media at high and low ionic strength, respectively. Hypertonic conditions can occur mainly as hypernatreamia and hyperglycemia, often connected with dehydration, intake of salt or sugar in excess, hyperglycemic osmotic diuresis and acute renal failure. Therefore, drug investigation at hypertonic conditions is highly important in order to avoid additional destabilization of the cellular membranes and tissue damage [44].

The aim of our investigations was to obtain more information about mechanisms of interaction between saponin and the cellular membrane, which are behind the process of hemolysis with main focus on the influence of hypertonic conditions, which to the best of our knowledge is not investigated up to date. The hemolytic analysis was performed by spectrophotometrically applying two types of measurements: (1) end-point hemolysis (measurement of hemoglobin content in the supernatant) after incubation of erythrocytes in suspension media with different concentrations of saponin at a given tonicity; and (2) real-time measurements of hemolysis in media with different tonicity under the influence of the same concentration of saponin.

## 2. Results and Discussion

### 2.1. Hemolysis in NaCl Solutions at Different Osmotic Pressures

#### 2.1.1. End-Point Assays in NaCl Solutions

**Figure 1:**
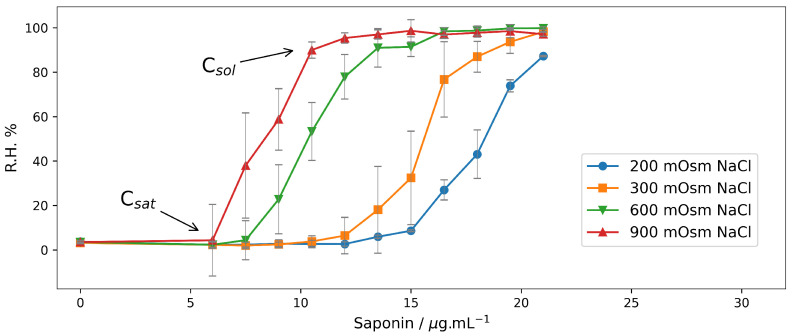
Relative hemolysis (R.H.%) induced by saponin in erythrocyte suspensions (Hct 0.5%) after 30 min incubation in NaCl solutions with different osmolarities. All solutions were buffered with 10 mM phosphate buffer pH 7.4. Light absorption of the supernatant was measured at wavelength λ = 416 nm. (*n* = 3).

The graphs in the Figure 1 show the concentration dependence of saponin-induced relative hemolysis (Equation (1)) for four various tonicities—200 mOsm; 300 mOsm, 600 mOsm and 900 mOsm. Here, NaCl determines the osmotic activity of the suspension medium.

All four curves in Figure 1 exhibit a sigmoidal shape, which is typical for the action of a hemolytic agent. Below some threshold concentration ($C_{sat}$) [45,46], the saponin does not cause any hemolysis. Above this concentration, the erythrocytes start to hemolyse. Above another value of the saponin concentration ($C_{sol}$) the RBCs are completely solubilized. The results for these threshold concentrations of saponin are presented in Table 1.

The graphs (in Figure 1) and the table data (Table 1) clearly show the higher hemolytic activity of saponin in a hypertonic solution and stabilization of the erythrocyte membranes under hypotonic conditions. For RBCs suspended in hypotonic medium (200 mOsm) hemolysis is measurable at saponin concentrations above 12 μg/mL. In a hypertonic solution (900 mOsm NaCl), hemolysis can be detected at a two-times lower concentration (6 μg/mL), whereas at 12 μg/mL, saponins in the suspension the RBCs are completely hemolysed.

#### 2.1.2. Real-Tme Assays in NaCl Solutions

**Figure 2:**
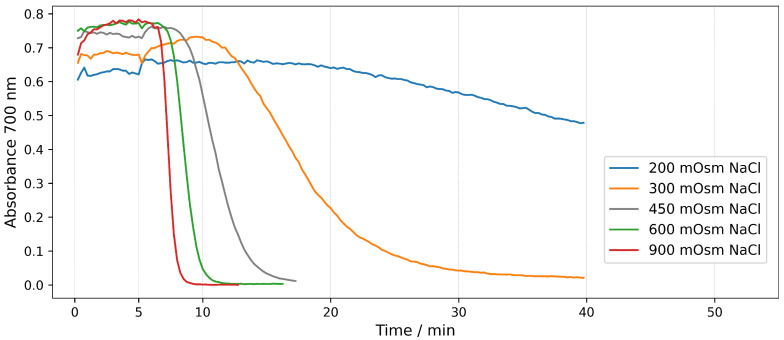
Time dependence of the absorbance at 700 nm during the hemolysis of erythrocyte suspension with Hct 0.07 % induced by 15 μg/mL saponin. All solutions were buffered with 10 mM phosphate buffer pH 7.4. Different curves correspond to indicated tonicities of the NaCl solutions. Saponin is added at the time point of 5 min.

The hemolysis of erythrocytes can also be monitored by measuring the light absorption of the whole suspension in real time without centrifugation. At wavelengths at which the hemoglobin does not absorb light, the values of the absorbance are due only to scattering of the light from the intact erythrocytes. During the course of hemolysis with the hemoglobin released from the cells, the light scattering decreases, which results in a decrease in the measured absorbance. The advantage of this type of hemolytic assay is that the hemolysis is quantified in situ at any moment of the process, i.e., it shows the kinetics of hemolysis in contrast to the end point values obtained in the case of ordinary hemolytic assays. Figure 2 shows hemolysis in real-time of erythrocyte suspensions with Hct of 0.07%. For all tonicities, the hemolysis was induced by 15 μg/mL saponin (final concentration in the solution). Saponin was added 5 min after placing the erythrocytes in the medium. Hemolysis was monitored in real time by recording the absorbance of the suspension at 700 nm.

It can be seen in Figure 2 that the shape of all curves in the graph has a sigmoidal character, which shows that hemolysis proceeds in a similar way, but the process takes different times at different tonicities. For all tonicities there is an initial lag period, after which the absorbance starts to decrease, indicating that hemolysis occurs. The duration of this initial period increases gradually with the decrease in the tonicity of the solution, from 2 min for the most hypertonic solution (900 mOsm) to 7 min for the physiological solution and to 15 min for the hypotonic solution. The speed of hemolysis is the highest in the most hypertonic solution (900 mOsm) and gradually decreases with decreasing the tonicity. Accordingly, the duration of the whole process increases with decreasing the tonicity. The hemolysis caused by the corresponding amount of saponin in the hypotonic solution is so slow that it does not proceed to the end for the duration of the experiment. Overall, the results show a clear dependence of the speed of hemolysis on the tonicity of the solution, i.e., hemolysis accelerates as the tonicity increases.

**Figure 3:**
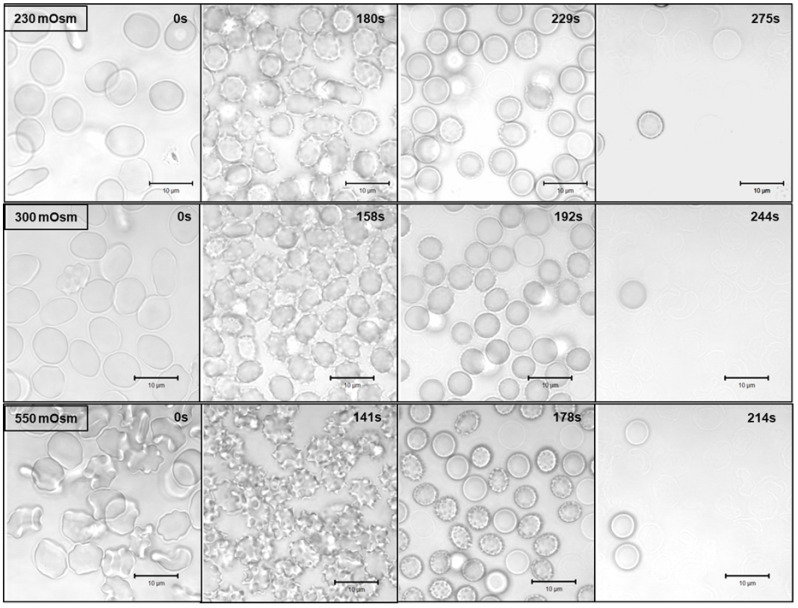
Representative confocal laser scanning micrographs of washed erythrocytes in 230 mOsm (upper panel), 300 mOsm (middle panel) and 550 mOsm (lower panel) of NaCl, 10 mM phosphate buffer, pH 7.4 supplemented with 2 % human serum albumin for shape stabilization. The images are selected from time series taken after addition of saponin (final concentration 300 μg/mL) directly under the microscope.

As mentioned earlier, the most probable mechanism of hemolysis caused by saponin is the colloid-osmotic mechanism. Since this mechanism is related to swelling of the cells and subsequent lysis, it seems strange that the cells in hypotonic solutions that are already osmotically swelled lyse slower than the shrunk cells in the hypertonic solutions. In order to confirm the colloid-osmotic mechanism, we used the time series option of a confocal microscope to record the shape and volume changes of the erythrocytes suspended in hypotonic (230 mOsm), isotonic (300 mOsm) and hypertonic (550 mOsm) solutions after the addition of 300 μg/mL saponin. Representative images at the time of significant shape change, start and end of the process of hemolysis are shown in Figure 3.

As it can be seen from the microscope observations, the initial shape of the red blood cells is different in hypotonic (230 mOsm), isotonic (300 mOsm) and hypertonic (550 mOsm) media. Before addition of saponin, the normal discoid shape is observed at the isotonic conditions. At 230 mOsm, hypotonic, the cells are slightly swollen, but still their shape is discoid. The situation changes at hypertonic conditions where the cells shrink and start to transform their shape into echinocytes. The addition of saponin causes, after a certain lag period, a shape change to echinocytes for all three osmolarities followed by swelling, spherocyte formation and finally by hemolysis. Interestingly, the lag period depends on the osmolarity of the medium being the shortest at hypertonic conditions and the longest at hypotonic conditions. These observations correlate well with the results obtained by the real time hemolysis assays, where the lag period was also decreasing with increasing osmolarity of the medium. However, the samples prepared for time series with the confocal microscope we applied a much higher saponin concentration (300 μg/mL vs. 15 μg/mL) to achieve faster hemolysis and suitable observation times. The most valuable additional information from the microscopic observations is to note the changes that occur in the erythrocytes from the time of saponin addition until just before hemolysis. It is clearly seen that there is no membrane solubilization or vesicle formation. The swelling, especially at isotonic and hypertonic conditions, can only be explained as a colloid-osmotic process initiated by the formation of pores in the lipid bilayer of the cell membrane.

### 2.2. Hemolysis in Mannitol/NaCl Solutions at Different Osmotic Pressures

In some works [25], it is stated that the reduced thickness of the electric double layer caused by higher ionic strength could contribute to stronger adsorption and easier incorporation of some detergents into the cell membrane and faster hemolysis, respectively. In order to study the effect of the thickness of the electric double layer on the rate of hemolysis, we performed a series of experiments in which erythrocytes were suspended in media with different tonicities, but with the same ionic strength. The different osmolarity was due to different concentrations of mannitol, and the same ionic strength was achieved with the same concentration of NaCl and the buffer.

#### 2.2.1. End-Point Assays in Mannitol/NaCl Solutions

**Figure 4:**
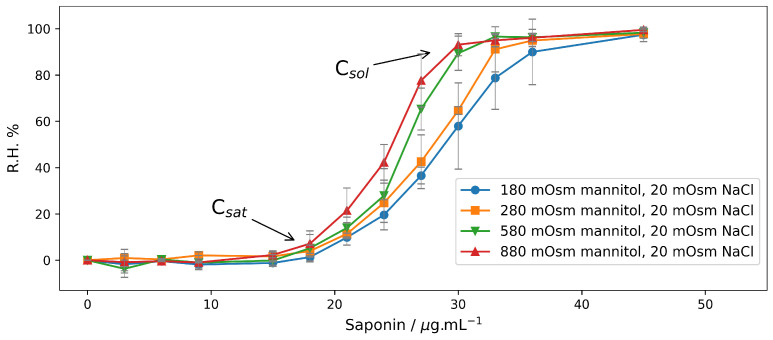
Relative hemolysis (R.H. %) induced by saponin in erythrocyte suspension (Hct 0.5%) after 30 min incubation in mannitol/NaCl solutions with different osmolarities. All solutions were buffered with 10 mM phosphate buffer pH 7.4. Light absorption of the supernatant was measured at wavelength λ = 416 nm. (*n* = 3).

Results with end-point hemolysis are presented in Figure 4. Qualitatively, the results in Figure 4 do not differ from those in Figure 1. Even in solutions with the same ionic strength (Figure 4), hemolysis occurs more easily under hypertonic conditions, and the hypotonic solution further stabilizes the cells, as it is in solutions with different ionic strength (Figure 1). On the other hand, it is evident that the end-point hemolysis curves are shifted to higher concentrations of saponin in the presence of mannitol, i.e., mannitol has a membrane-stabilizing-like effect. This is in accordance with the results of Winter [47], who demonstrated that in an isotonic medium, increasing the concentration of sucrose or glycine suppressed the Saponin-induced hemolysis. In our case, the concentration at which hemolysis started ($C_{sat}$) was much higher than when the erythrocytes were incubated in NaCl. It is interesting to note that there is no significant difference between the values of $C_{sat}$ (Figure 4) for the different tonicities, while the values of $C_{sol}$ differ more prominently.

#### 2.2.2. Real-Time Assays in Mannitol/NaCl Solutions

**Figure 5:**
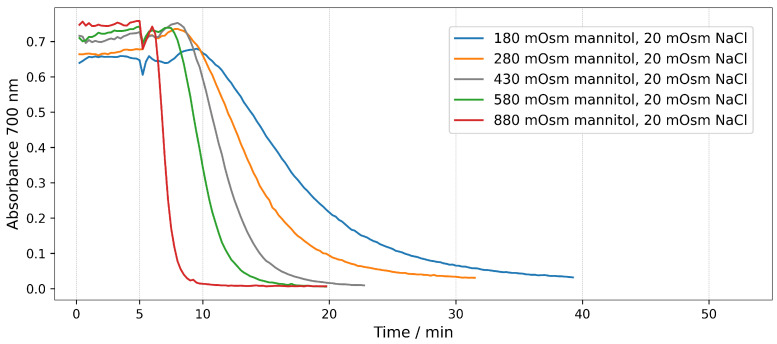
Time dependence of the absorbance at 700 nm during the hemolysis of erythrocyte suspension with Hct 0.07 % induced by 30 μg/mL saponin. Different curves correspond to indicated tonicities of the mannitol/NaCl solutions. All solutions were buffered with 10 mM phosphate buffer pH 7.4. Saponin is added at the time point of 5 min.

To confirm the results of end-point hemolytic assays, hemolysis was measured in real time, as well. The results in Figure 5 show that at constant ionic strength, the saponin induces faster hemolysis in hypertonic media. In contrast to Figure 2, the results in Figure 5 are obtained with a two times higher concentration of the saponin, in order to achieve a similar rate of hemolysis.

**Figure 6:**
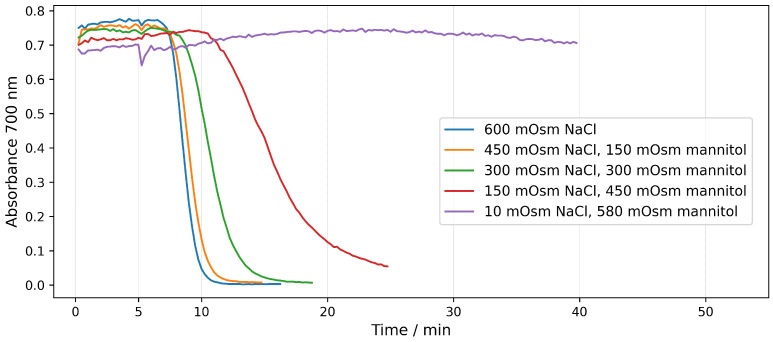
Time dependence of the absorbance at 700 nm during the hemolysis of erythrocyte suspension with Hct 0.07% induced by 15 μg/mL saponin. Different curves correspond to the same total osmolarity but different content of NaCl and mannitol. Saponin is added at the time point of 5 min.

In order to directly compare the action of NaCl and mannitol, we performed an experiment with the same osmolarity, but with different complementary contents of NaCl and mannitol (Figure 6). In these experiments, NaCl was gradually replaced by mannitol with a step of 150 mOsm. It can be seen from the graph that the gradual replacement of NaCl with mannitol slows down the hemolysis.

Two main mechanisms of hemolysis from detergents are indicated in the research papers [25,27]—colloid-osmotic and solubilization with the formation of micelles from the detergent and membrane lipids. The results presented in Figure 6 once again support the claim that the mechanism of hemolysis at the saponin concentrations used is colloid-osmotic and depends on the type of particles that penetrate the erythrocytes after the formation of defects/pores. If the mechanism were solubilization, hemolysis would proceed at the same rate regardless of the ratio of NaCl and mannitol.

The presented results can be explained by two processes—the incorporation of saponin in the outer monolayer of the erythrocytes membrane and the diffusion of solutes through defects in the membrane.

It is well known that saponins are amphiphilic compounds that adsorb in the outer monolayer of the membrane lipid bilayer and subsequently bind mainly to cholesterol and some membrane lipids (in lipid rafts) [40]. As a result, complexation of cholesterol with saponin occurs, which causes the appearance of membrane defects like pores in the lipid bilayer [33] with a diameter that gradually increases to finally reach the size of 4–5 nm [31,34]. The presence of such defects in the membrane leads to changes in the permeability of the membrane.

The diameter of hemoglobin at physiological pH is about 5.5 nm [48], so only ions and small molecules can diffuse trough the membrane. According to different estimations, the radius of sodium ions in a hydrated state is 0.358 nm, of potassium ions is 0.331 and of chloride ions is 0.332 nm [49].

The initial change of the shape of erythrocytes to become echinocytes can be explained by the two above-mentioned processes. It is known that adsorption of surfactants or amphiphilic drugs in the outer monolayer of erythrocytes increases the area of the latter, which leads to the formation of echinocytes [27]. Other authors had shown by molecular dynamics simulations that the saponin molecules should accumulate in the lipid rafts in the outer monolayer and create a convex curvature of the membrane at the site of accumulation [40].

Another process that can contribute to the formation of echinocytic shape would be an initial leakage of potassium ions out of the cell through the membrane defects. The diffusion of the potassium cations out of the cell prevails the diffusion of sodium ions into the cell because of the smaller size and greater diffusion coefficient of the potassium ion compared to those of the sodium ion [50]. This initial diffusion of the potassium ions out of the cell decreases the osmotic pressure inside the cell that leads to the shrinkage of the cell and the development of the echinocytic shape.

During the next stage, the sodium ions can pass freely through the defects in the membrane driven by their concentration gradients. As the concentration of ions and small molecules equalize on both sides of the membrane, the colloid osmotic pressure becomes higher inside the cell due to the hemoglobin that cannot diffuse out of the cell. The higher colloid osmotic pressure inside the cells leads to their swelling and lysis.

Dispersed in a hypertonic environment, saponin-treated cells lyse rapidly, as sodium ions from the outside will rapidly diffuse into the cells due to the greater concentration gradient through the membrane.

In contrast to inorganic ions, the diameter of the mannitol molecule is larger, with a radius of gyration of 0.417 nm [51], and diffusion into cells begins after the formation of larger pores in the membrane. As a result, the cells swell and lyse more slowly when NaCl is replaced with mannitol.

## 3. Materials and Methods

### 3.1. Materials

Saponin weiss rein (white pure, from quillaja bark) was purchased from Merck, Darmstadt, Germany. This saponin is often used for the preparation of standard solutions for the determination of saponin content in plant extracts [52,53].

D-mannitol and sodium chloride were purchased from Sigma-ALDRICH, St. Louis, MO, USA.

Venous blood from healthy volunteer donors was taken in a clinical laboratory of the University hospital of Trakia University, Stara Zagora, Bulgaria, on the day of investigation, conducted with the informed consent of patients in accordance with the Declaration of Helsinki and the protocol No 10/5 June 2019 of Ethic Cmmission of the Medical Faculty, Trakia University, Stara Zagora, Bulgaria.

### 3.2. Isolation of Erythrocytes

The erythrocytes were isolated from the blood plasma by centrifugation at 1700× *g* for 3 min. The upper layer of white blood cells was removed. The erythrocytes were washed two times in 150 mM NaCl. The initial suspension for further experiments contained washed erythrocytes in saline (150 mM NaCl) with hematocrit (Hct) 10%.

### 3.3. Assays of Hemolysis Caused by Saponin

#### 3.3.1. End-Point Hemolysis Assays

End-point hemolysis assays were performed in suspensions of erythrocytes with a Hct of 0.5% achieved by dilution at ratio 1/20 of the initial suspension of washed erythrocytes with solutions of different osmotic pressures—from hypotonic 200 mOsm to hypertonic 900 mOsm. In all experiments, the medium was buffered at pH = 7.4 with the same amount of 10 mM phosphate buffer. The osmotic pressure in the solution was maintained by the concentrations of NaCl and/or mannitol, and saponin was added at various final concentrations as shown in Table 2.

The erythrocytes were suspended in each medium and incubated for 30 min at room temperature. The samples were then centrifuged at 1700× *g* for 3 min and the hemoglobin concentration in the supernatant was determined spectrophotometrically by the absorption at 416 nm in a 1 mm cuvette (spectrophotometer Cary 60 UV-Vis, Agilent Technologies, U.S.). The relative hemolysis in each sample has calculated using Equation (1):

(1) $$R.H.=\frac{A_{i}-A_{0}}{A_{100}-A_{0}}\;\cdot \;100\%$$

where $A_{i}$ is the absorbance of the sample, $A_{0}$ is the absorbance of the mechanical hemolysis control in the corresponding tonicity solution without detergent, and $A_{100}$ is the absorbance of the 100 % hemolysis control, i.e., erythrocytes in water.

We also performed measurements of the absorption spectrum of saponin in order to show that saponin does not absorb light at 416 nm, as well as, at 700 nm, and that its presence in the solution does not influence the estimation of relative hemolysis. These data are added as supplementary material (Figure S1).

#### 3.3.2. Real-Time Hemolysis Assays

Real-time hemolysis was performed in suspensions of erythrocytes with a hematocrit of 0.067% with different osmotic pressure of the medium—from hypotonic 200 mOsm to hypertonic 900 mOsM. In all experiments, the medium was buffered at pH = 7.4 with the same amount of 10 mM phosphate buffer. The osmotic pressure in the solution was defined by the concentrations of NaCl and/or mannitol as shown in Table 3. Hemolysis was induced by addition of saponin at the 5-th min (final concentrations shown in Table 3) and was followed in real time by measuring the absorbance of the suspension at 700 nm in a 10 mm cuvette. At the selected wavelength, hemoglobin has no absorption peak and the absorption corresponds to light scattering by the intact erythrocytes. During hemolysis, the scattering decreases due to the release of hemoglobin.

As depicted on Table 3, three groups of experiments were performed.

The first group of experiments included hemolysis in NaCl solutions. Saponin was pipetted 5 min after suspending the erythrocytes in the medium, allowing them to reach a shape/volume equilibrium at the given osmolarity. The final concentration of saponin was 15 μLg/mL.

The second group of experiments included hemolysis of erythrocytes suspended in mannitol media of equal low ionic strength and different osmolarity. Saponin with a final concentration of 30 μLg/mL was added 5 min after the erythrocytes were suspended in the medium.

In the third group of experiments, hemolysis was investigated at an equal osmolarity of the medium (600 mOsm), but with a gradual replacement of NaCl by mannitol (Table 3). Saponin with final concentration 15 μg/mL was added 5 min after the erythrocytes were suspended in the medium.

We also performed real-time measurements of erythrocyte suspensions in different tonicities and content without saponin in order to show that in these solutions there is no hemolysis if saponin is not present. The data are added as supplementary material (Figures S2 and S3).

### 3.4. Confocal Laser Scanning Microscopy (CLSM)

LSM 510 Meta (Carl Zeiss AG, Oberkochen, Germany) confocal laser scanning microscope with 100× oil-immersion objective (numerical aperture 1.3) was used for the live imaging of the hemolysis process applying the time series mode of the software. Erythrocyte suspensions were prepared in 230 mOsm, 300 mOsm and 550 mOsm NaCl solutions supplemented with 2 % human serum albumin (HSA) for shape stabilization. A 20 μL drop of each suspension was placed on a cover slip and 2 µL of a 0.3 % saponin solution was carefully added shortly after the start of the time series. The samples were scanned every second until the process of hemolysis was completed. The images were obtained with the 488 nm line of an argon laser in transmission mode.

## 4. Conclusions

From our results, it can be concluded that the effect of saponin on the hemolysis of erythrocytes depends strongly on the osmolarity of the solution. In the investigated range from 200 mOsm to 900 mOsm, the saponin is most effective as a hemolytic agent at the most hypertonic conditions and its effect gradually decreases with the decrease in osmolarity. This dependence is accessed by both types of hemolytic assays used in the current investigation, end-point hemolytic assay and real-time hemolytic assay. In end-point hemolytic assays, the critical concentrations above which RBCs start to haemolyse, as well as, the critical concentrations above which RBCs are completely solubilized decrease with the increase in osmolarity. Real-time hemolytic assays show that the greater the osmolarity, the faster the hemolysis process. The dependence of the saponin hemolytic effect on osmolarity is more pronounced in pure salt solutions than in solutions with osmotic pressure regulated by mannitol. At the investigated conditions and saponin concentrations, the hemolysis of RBCs proceeds by a colloid-osmotic mechanism. We hypothesize that the kinetics of the process is defined by the dimensions and diffusion coefficients of the solute that regulate the osmotic pressure.

## Figures and Tables

**Table 1:** Values of saponin threshold concentrations in NaCl solutions obtained from Figure 1. $C_{sat}$ is the lowest concentration of saponin at which hemolysis is detectable and $C_{sol}$ is the lowest saponin concentration at which the process of hemolysis is completed.

NaCl, mOsm	$C_{\mathrm{sat}}$, μg/mL	$C_{\mathrm{sol}}$, μg/mL
200	12	>21
300	10.5	21
600	7.5	15
900	6	12

**Table 2:** Summary of experimental sets and composition of solutions applied in the measurements of end-point hemolysis. All solutions were buffered with 10 mM phosphate buffer pH 7.4.

Osmotic Pressure	NaCl	Manitol	Saponin
mOsm	mM	mM	μg/mL
Hemolysis in NaCl solutions			
200	100	-	
300	150	-	0 ÷ 21
600	300	-	Step 1.5
900	450	-	
Hemolysis in manitol at low ionic strength			
200	10	180	
300	10	280	0 ÷ 45
600	10	580	Step 3.0
900	10	880	

**Table 3:** Summary of experimental sets and composition of solutions applied in the measurements of real time hemolysis. All solutions were buffered with 10 mM phosphate buffer pH 7.4.

Osmotic Pressure	NaCl	Manitol	Saponin
mOsm	mM	mM	μg/mL
Hemolysis in NaCl solutions			
200	100	-	15
300	150	-	
450	225	-	
600	300	-	
900	450	-	
Hemolysis in manitol at low ionic strength			
200	10	180	30
300	10	280	
450	10	430	
600	10	580	
900	10	880	
Hemolysis in NaCl/manitol solutions			
600	300	0	15
600	225	150	
600	150	300	
600	75	450	
600	10	580	

## Data Availability

The data are available upon request to the authors.

## Supplementary Materials

The following supporting information can be downloaded at: https://www.mdpi.com/article/10.3390/molecules28207096/s1, Figure S1. Absorption spectra of saponin in 150 mM NaCl solution buffered with phosphate buffer to pH 7.4. The saponin concentration is given in the legend. Optical pathlength 10 mm. Figure S2. Time dependence of the absorbance at 700 nm of erythrocyte suspension with Hct 0.07% in NaCl solutions. All solutions were buffered with 10 mM phosphate buffer pH 7.4. Different curves correspond to indicated tonicities of the NaCl solutions. No saponin is added at the solution and no hemolysis is detected. Figure S3. Time dependence of the absorbance at 700 nm of erythrocyte suspension with Hct 0.07% in mannitol/NaCl solutions. All solutions were buffered with 10 mM phosphate buffer pH 7.4. Different curves correspond to indicated tonicities. No saponin is added at the solution and no hemolysis is detected.

## Abbreviations

The following abbreviations are used in this manuscript:

RBCs	Red Blood Cells
Hct	Hematocrit
R.H.	Relative hemolysis
