# Supplementary material for: Hemolysis by Saponin Is Accelerated at Hypertonic Conditions
Source: Molecules. 2023 Oct 15;28(20):7096. doi: 10.3390/molecules28207096 (PMC10609376; doi:10.3390/molecules28207096)
Supplement: Supplementary file 1 [file molecules-28-07096-s001.zip › molecules-2611607-supplementary.pdf]

Supplementary material

Hemolysis by Saponin is Accelerated at Hypertonic Conditions

Boyana Paarvanova , Bilyana Tacheva, Gergana Savova, Miroslav Karabaliev and Radostina Georgieva

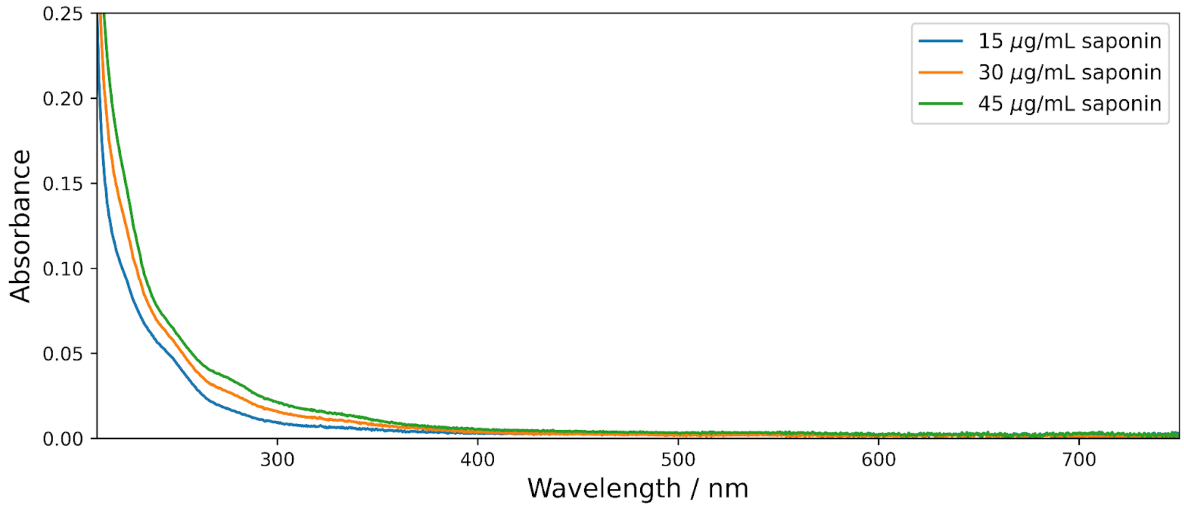

Figure S1. Absorption spectra of saponin in 150 mM NaCl solution buffered with phosphate buffer to pH 7.4. The saponin concentration is given in the legend. Optical pathlength 10 mm.

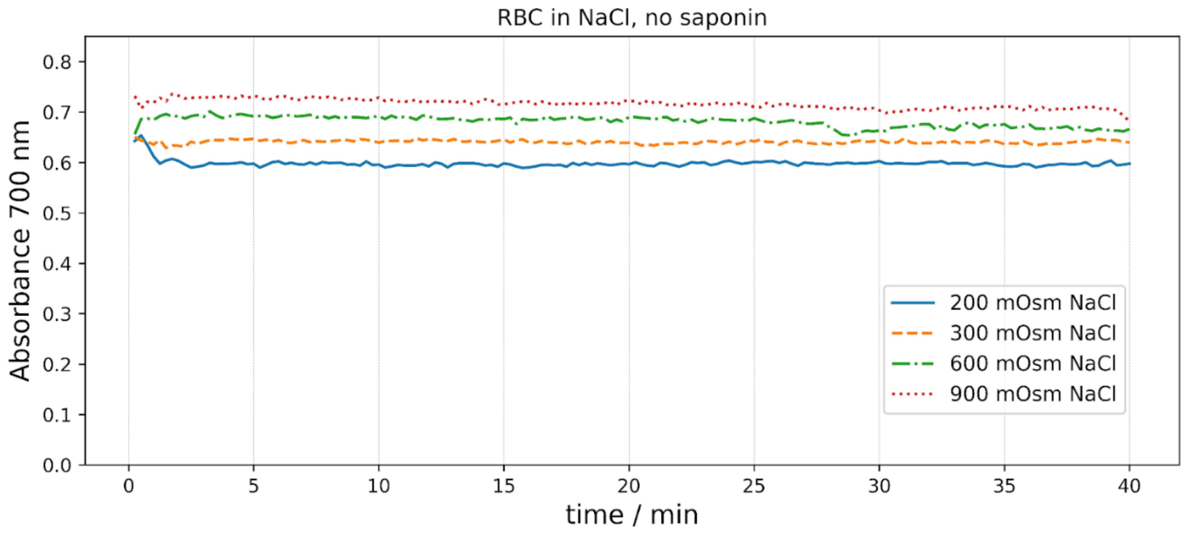

Figure S2. Time dependence of the absorbance at 700 nm of erythrocyte suspension with Hct 0.07 % in NaCl. solutions All solutions were buffered with 10 mM phosphate buffer pH 7.4. Different curves correspond to indicated tonicities of the NaCl solutions. No saponin is added at the solution and no hemolysis is detected.

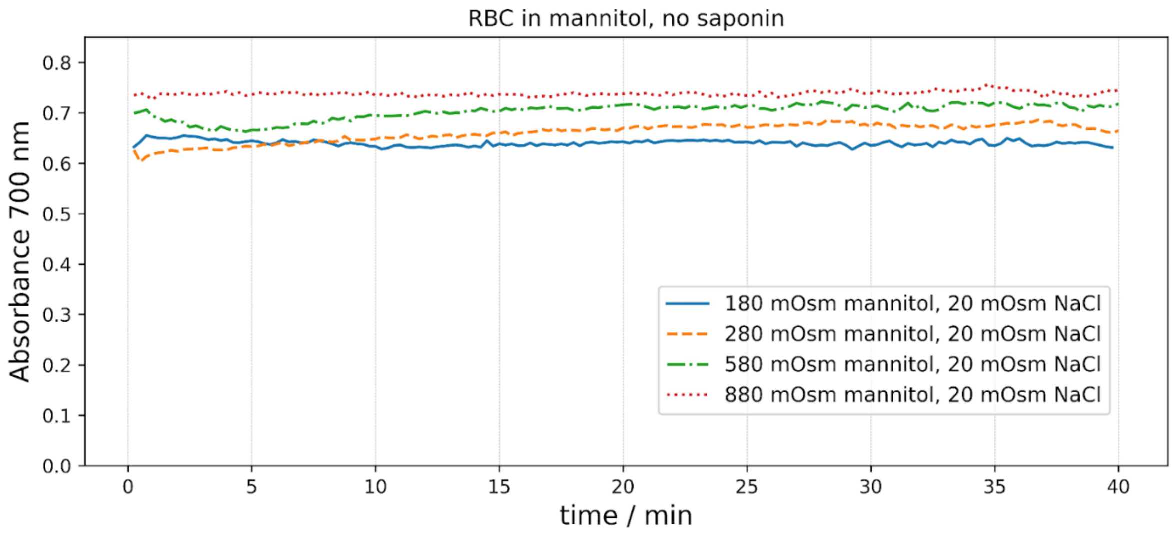

Figure S3. Time dependence of the absorbance at 700 nm of erythrocyte suspension with Hct 0.07 % in mannitol/NaCl solutions. All solutions were buffered with 10 mM phosphate buffer pH 7.4. Different curves correspond to indicated tonicities. No saponin is added at the solution and no hemolysis is detected.
